# Rational development of transformation in *Clostridium thermocellum* ATCC 27405 via complete methylome analysis and evasion of native restriction–modification systems

**DOI:** 10.1007/s10295-019-02218-x

**Published:** 2019-07-24

**Authors:** Lauren A. Riley, Lexiang Ji, Robert J. Schmitz, Janet Westpheling, Adam M. Guss

**Affiliations:** 1 Bredesen Center, University of Tennessee, Knoxville, TN 37996 USA; 2 Biosciences Division, Oak Ridge National Laboratory, Oak Ridge, TN 37830 USA; 3 Institute of Bioinformatics, University of Georgia, Athens, GA 30602 USA; 4 Department of Genetics, University of Georgia, Athens, GA 30602 USA

**Keywords:** *Clostridium thermocellum*, Restriction–modification systems, Whole-genome bisulfite sequencing, Single molecule, Real-time sequencing, Methylome

## Abstract

A major barrier to both metabolic engineering and fundamental biological studies is the lack of genetic tools in most microorganisms. One example is *Clostridium thermocellum* ATCC 27405^T^, where genetic tools are not available to help validate decades of hypotheses. A significant barrier to DNA transformation is restriction–modification systems, which defend against foreign DNA methylated differently than the host. To determine the active restriction–modification systems in this strain, we performed complete methylome analysis via single-molecule, real-time sequencing to detect 6-methyladenine and 4-methylcytosine and the rarely used whole-genome bisulfite sequencing to detect 5-methylcytosine. Multiple active systems were identified, and corresponding DNA methyltransferases were expressed from the *Escherichia coli* chromosome to mimic the *C. thermocellum* methylome. Plasmid methylation was experimentally validated and successfully electroporated into *C. thermocellum* ATCC 27405. This combined approach enabled genetic modification of the *C. thermocellum*-type strain and acts as a blueprint for transformation of other non-model microorganisms.

**Electronic supplementary material:**

The online version of this article (10.1007/s10295-019-02218-x) contains supplementary material, which is available to authorized users.

## Introduction

Most microbial metabolic engineering for biotechnology research is performed in model organisms, because they are well studied and have a large toolbox to enable genetic modifications [7]. Non-model organisms, on the other hand, are an attractive alternative as potential industrial platforms for bioconversion, because they often possess complex phenotypes that are not currently feasible to engineer into model organisms, such as the ability to grow at extreme pH, extreme robustness/toxicity tolerance, or the ability to catabolize less common substrates such as syngas, methane, cellulose, or lignin [49, 52]. However, work with non-model organisms is limited due to a lack tools and knowledge of the organism. A major barrier to genetic tools development is the inability to efficiently transform DNA, and to routinely allow for the use of non-model organisms; a systematic process for developing transformation is needed.

One of the major barriers to successful transformation of bacteria is native DNA restriction–modification (RM) systems, which act as a bacterial immune system to cut DNA that is methylated differently than the host [2]. RM systems are classified into four types. In Types I, II, and III, a restriction enzyme typically cuts DNA that is unmethylated at a specific recognition sequence, and a corresponding DNA methyltransferase adds a methyl group to the host’s DNA to protect these same sequences from restriction enzyme activity [2]. Type I systems are comprised of three subunits: a DNA methyltransferase, a DNA recognition subunit, and a restriction enzyme. This type of system recognizes two motifs of 3–4 bases separated by any 5–8 bases [25], such as the EcoKI system that targets A**A**CNNNNNNG**T**GC (N is any base), and motifs are methylated at the N-6 position of one adenine per DNA strand to form *N*^6^-methyladenine (m^6^A). Type II and III systems are typically comprised of a methyltransferase and a restriction enzyme subunit. Type II systems are largely studied and commonly used as tools in molecular biology. Their recognition systems are often palindromic, and they can methylate bases to form m^6^A, *N*^4^-methylcytosine (m^4^C), or 5-methylcytosine (m^5^C) [16, 31]. Type III systems recognize non-palindromic motifs and typically methylate to form m^6^A [38]. The last group, Type IV, is only comprised of a restriction enzyme, which recognizes motifs that are methylated differentially than the host [19, 38].

To successfully evade most native RM systems, DNA needs to be methylated in the same way as the target organism, and many studies have shown that overcoming these systems is important for efficient DNA transformation [4, 23, 33, 41, 50]. To rationally evade RM systems, the targeted motifs first need to be identified. Microbes protect their chromosomal DNA from restriction via DNA methylation; therefore, methylome analysis can be used to identify these motifs. While not all DNA methyltransferases are associated with restriction enzymes [3], methylome analysis does reveal all potentially active Type I, II, and III RM systems present in an organism. A common approach to microbial methylome analysis uses single-molecule real-time (SMRT) sequencing on the PacBio platform [9, 17, 24, 28], which identifies m^6^A and m^4^C motifs based on kinetic delays in nucleotide incorporation when a base is methylated [37]. This approach has been specifically used to help overcome restriction barriers to genetic transformation in a number of organisms [4, 34, 41]. SMRT sequencing can also identify m^5^C motifs, but the signal is less strong, so it requires immense coverage of the genome or modification of the methylated base via Tet1 oxidation [5], approaches that are not common practice. An alternate approach to detect m^5^C and m^4^C is methyl-C sequencing for whole-genome bisulfite sequencing (WGBS), in which the cytosine (but not m^5^C) is deaminated to uracil, followed by polymerase chain reaction (PCR) to convert the resulting uracils to thymines. The resulting PCR-amplified DNA can then be sequenced, and remaining cytosines in the sequence were previously methylated. This approach has not been routinely used in bacteria, and we have only identified a few studies that utilized WGBS for bacterial methylome analysis for characterization of RM systems [13, 44, 51].

*Clostridium thermocellum* is an anaerobic, thermophilic bacterium that efficiently deconstructs lignocellulosic biomass via large cell surface-associated enzyme complexes called cellulosomes. The resulting soluble sugars are fermented into products such as organic acids and ethanol [1]. There is particular interest in *C. thermocellum* due to its potential for biofuel production from lignocellulose via a process called consolidated bioprocessing (CBP), in which cellulolytic enzyme production and fermentation occur in a single reactor [26, 27]. The cellulosome was first discovered in the *Clostiridium thermocellum-*type strain, ATCC 27405 [15], and a substantial amount of work has been done on this strain to understand carbon metabolism and the genes related to cellulosome production [8, 32, 35, 43, 48]. All of these studies examined the wild-type strain using omics tools such as transcriptomics and proteomics, but to date, this strain has not been genetically modified. This is in stark contrast to *C. thermocellum* DSM 1313, where transformation and genetics are readily available [29] and extensive metabolic engineering has been achieved [18, 30, 40, 45, 46].

The lack of genetic tools in strain ATCC 27405 has hindered both fundamental studies and development of this strain for bioengineering. Thus far, one publication has demonstrated transformation of this strain using a custom-made electroporator [47], and additional attempts to transform *C. thermocellum* ATCC 27405 using commercially available equipment have been unsuccessful. One other publication demonstrated a low transformation rate of 2.5 ± 1.5 colonies per microgram of DNA with a single plasmid using large cell and DNA volumes, which makes the process hard to utilize in the future [20].

One possible reason for the difference in success of transformation between strains DSM 1313 and ATCC 27405 is differences in RM systems, which are understudied in *C. thermocellum* ATCC 27405. New England Biolabs Restriction Enzyme Database (REBASE) [39] predicts the RM systems encoded in both strains of *C. thermocellum*, where ATCC 27405 encodes eight restriction systems and DSM 1313 only encodes five. Of the eight potential RM systems in ATCC 27405, one is a putative Type I system, genes Cthe_1144–1145, though it seems to be missing the restriction enzyme subunit. There are six putative Type II systems encoded: Cthe_1511–1513, Cthe_1638–1639, Cthe_1748–1749, Cthe_2470–2471, Cthe_2319–2320, and Cthe_1748–1749. Cthe_2740 and Cthe_1728–1729 do not have annotated associated restriction enzymes, but the other four encode putative restriction enzymes do. The genome also encodes one Type III RM system, Cthe_0518-0519, with Cthe_0518 being a predicted restriction enzyme. No apparent Type IV systems are encoded in the genome. Previously, one of these RM systems was shown to be active in cell extracts of strain ATCC 27405, and extracts exhibited endonuclease activity targeting the motif G**A**TC, similar to the *Mbo*I restriction enzyme [14]. Here, we report the methylome of *C. thermocellum* ATCC 27405, identify and express the active methyltransferases, validate the expression for in vivo methylation, and successfully transform *C. thermocellum* ATCC 27405.

## Materials and methods

### Strains, plasmids, and growth conditions

*Escherichia coli* Top 10 Δ*dcm::frt* was created by deleting *dcm* in Top10 (Invitrogen) with the lambda Red recombinase system as previously described [6]. *E. coli* strains were grown aerobically in LB at 37 °C, and chloramphenicol was added for plasmid selection at a final concentration of 15 µg/mL. *C*. *thermocellum* ATCC 27405 was grown in CTFUD medium [40], which is comprised of (per L) 3 g sodium citrate tribasic dehydrate, 1.3 g ammonium sulfate, 1.43 g potassium phosphate monobasic, 1.8 g potassium phosphate dibasic trihydrate, 0.5 g cysteine HCL, 10.5 g MOPS sodium salt, 6 g glycerol-2-phosphate disodium, 5 g cellobiose, 4.5 g yeast extract, 0.13 g calcium chloride dehydrate, 2.6 g magnesium chloride hexahydrate, 0.0001 g ferrous sulfate heptahydrate, and 0.5 ml 0.2% (w/v) resazurin. The pH is adjusted to 7.0 after addition of MOPS with 45% (w/v) potassium hydroxide. *C. thermocellum* was grown at 50 or 55 °C, as indicated, in a Coy anaerobic chamber. Thiamphenicol was added to the medium when appropriate at a final concentration of 5 µg/mL.

Two plasmids, pNJ020 and pAMG216, were used for transformation of *C. thermocellum*, each containing the pNW33N origin of replication for *C. thermocellum* and the *cat* gene for thiamphenicol selection. Plasmid pNJ020 contains the p15a origin for medium copy-number replication in *E. coli*. Plasmid pAMG216 is derived from pAMG205 with the yeast machinery deleted, and it has a high copy-number pUC origin of replication for *E. coli* [11]. Methyltransferases were codon optimized for *E. coli* and synthesized with the T5Lac promoter by GenScript Biotech Corp (New Jersey, USA), amplified by PCR, and cloned into CRIM integration vectors [12] using Gibson assembly (New England Biolabs, NEB). The native *C. thermocellum* gene Cthe_0519 was cloned into pAH55 [12]. The bifunctional methyltransferase phi3TI, from *Bacillus* phage phi3T, was cloned into pAH144. Each plasmid was integrated, using the CRIM system [12], into the *λ* and HK022 phage *attB* sites of *E. coli* Top 10 *Δdcm::frt*, resulting in strain AG2006 (genotype: Top10 *Δdcm::frt* λ::Cthe_0519 HK022::phi3TI). Complete, annotated plasmid sequences are available in Supplemental File 1.

### SMRT sequencing methylome analysis

Genomic DNA from *C. thermocellum* ATCC 27405 was isolated using the Genomic Tip kit (Qiagen according to the manufacturer’s instructions and sent to Expression Analysis (Durham, NC, USA) for sequencing on a Pacific Biosciences (PacBio) instrument. Single-molecule real-time (SMRT) sequencing was performed using the PacBio RS technology with four SMRT cells. Methylated sequences were determined by Expression Analysis using the SMRT Analysis software [10].

### Whole-genome bisulfite sequencing analysis

MethylC-seq libraries were prepared and Illumina sequencing was performed (Genomics & Bioinformatics Core, University of Georgia) using an Illumina NextSeq 500 instrument. For data processing, raw reads were trimmed for adapters and preprocessed to remove low-quality reads using cutadapt 1.9.dev1 [21]. Qualified reads were aligned to the *C. thermocellum* ATCC 27405 reference genome [42]. Fully unmethylated lambda DNA was used as a control to calculate the sodium bisulfite reaction non-conversion rate of unmodified cytosines. Only cytosine sites with a minimum coverage (set as 3) were selected for subsequent analysis. Binomial test coupled with Benjamini–Hochberg correction was adopted to determine the methylation status of each cytosine. The m^5^C motifs were identified as previously described in [51].

### Determining functionality of the methyltransferases

Plasmids pNJ020 and pAMG216 were transformed into *E. coli* strains AG2006 and Top10 *Δdcm::frt* and grown in liquid culture with 0.1 mM IPTG to induce methyltransferase expression. Plasmid pNJ020 was isolated and digested in three separate reactions with *Nla*III, *Tse*I and *Hin*dIII, and *Hae*III and *Hin*dIII (New England Biolabs, Ipswich, MA, USA) according to the manufacturer’s instructions. To determine the amount of DNA methylated, digested plasmid was separated via agarose gel electrophoresis and quantified using BioRad Gel Imager software.

### Transformation of *C. thermocellum* ATCC 27405

Three 5 mL cultures were inoculated with *C. thermocellum* ATCC 27405 and grown overnight. Three 500 mL cultures were inoculated with 1% of the overnight cultures and grown at 55 °C. Cultures were harvested at an optical density (O.D.) of ~ 1.0, transferred to a 500 mL centrifuge bottle, placed on ice for 30 min, and then centrifuged at 4 °C at 5000×*g* for 15 min. The supernatant was decanted and cells were washed with 250 mL cold electroporation buffer (10% glycerol, 250 mM sucrose), which was added without disrupting/resuspending the cell pellet. Cells were spun again, and the wash was repeated two more times. After the last wash, the electroporation buffer was completely removed, and the cell pellets were resuspended in 200 µL electroporation buffer and transferred to a microcentrifuge tube. Using fresh electrocompetent cells from each batch, 20 µL of cells were transformed with 1 µg of DNA. Square wave electroporation was performed in a 1 mM electroporation cuvette at 1200 v with a 1.5 ms pulse. Cells were resuspended in 1 mL of CTFUD liquid medium and mixed with molten CTFUD supplemented with 1.5% agar and thiamphenicol, allowed to solidified, and incubated at 50 °C for 3–5 days, when colonies were counted.

## Results

### Complete methylome analysis of *C. thermocellum* ATCC 27405

To determine which of the eight RM systems, predicted by REBASE, are active, methylome analysis was performed. All methylated motifs in *C. thermocellum* ATCC 27405 were determined through two sequencing techniques, PacBio SMRT sequencing and MethylC-sequencing for WGBS. PacBio SMRT sequencing detected three m^6^A motifs and WGBS detected two m^5^C motifs (Table 1). Based on the type of motif and homology to known enzymes, each motif was tentatively assigned to a DNA methyltransferase (Table 1). *Cthe_2470* and *Cthe_1511* are both annotated as Dam methyltransferases, and *Cthe_1511* is encoded in a putative operon with the *Mbo*I-type restriction enzyme, suggesting that these enzymes target G**AT**C. The CNC**A**NNNNNN**T**TC motif is consistent with Type I RM system motifs (two 3–4 base-specific sites separated by 5–8 Ns), and so this is presumably targeted by the only Type I enzyme encoded in the genome—Cthe1144–1145. The non-palindromic sequence GTCAT is consistent with a Type III system, and so is likely targeted by the only encoded Type III enzyme, Cthe_0519, which is experimentally validated below. Cthe_2320 is annotated as a *Hae*III family restriction endonuclease, which targets G**GC**C, suggesting that *Cthe_2321* methylates G**GC**C. The only remaining m^5^C methyltransferase encoded in the genome is *Cthe_1749*, suggesting that it targets the remaining m^5^C site—GCWGC. These results are consistent with the predictions from REBASE.

**Table 1:** Methylated motifs in *C. thermocellum* ATCC27405

Motif	Type	% Modified	# Motifs in the genome	Predicted methyltransferase
G**AT**C	m^6^A	74	8234	*Cthe_2470* and *1511*
CNC**A**NNNNNN**T**TC	m^6^A	57.1	1775	*Cthe_1144*–*1145*
GTC**A**T	m^6^A	49.6	6945	*Cthe_0519*
G**C**W**G**C	m^5^C	100	6283	*Cthe_1749*
G**GC**C	m^5^C	100	12,192	*Cthe_2321*

Methylated bases are in bold. In cases where T or G are bold, the methylation is on the A or C of the complementary strand, respectively.  % m^6^A motifs were detected by PacBio SMRT sequencing, and m^5^C motifs were detected by WGBS. Modified is the percentage of these motifs in the genome that were detected as methylated. # of motifs in the genome is the number of times each motif appears in the genome. The “Predicted Methyltransferase” is the most likely *C. thermocellum* gene responsible for each methylation (*N* any base, *W* A or T)

### DNA methyltransferases were functionally expressed in *E. coli*

To methylate plasmid DNA in the same way as *C. thermocellum* ATCC 27405, we engineered *E. coli* to mimic the *C. thermocellum* ATCC 27405 methylome. One of the motifs, G**A**TC, is natively methylated by the Dam methyltransferase in *E. coli.* Two additional methyltransferases were expressed from the *E. coli* chromosome. First, Cthe_0519 was integrated the chromosome to target GTC**A**T. Next, a previously characterized bifunctional *Bacillus* phage methyltransferase, *phi3TI* [22], was added to the chromosome. The *Phi3TI* methyltransferase is known to methylate both G**C**W**G**C and G**GC**C, the same sites methylated in *C. thermocellum* by Cthe_1749 and 2321, so expressing this gene enabled methylation of both sites via the expression of a single gene. Cthe_0519 and *phi3TI* were added to the chromosome of an *E. coli dam*^+^*dcm*^−^ strain, so that Dam natively methylates GATC, a methylated motif observed in *C. thermocellum*, but Dcm does not methylate C**C**WGG, which was not seen in the *C. thermocellum* methylome analysis. The last methylated motif, CNC**A**NNNNNN**T**TC, does not occur on pNJ020, and therefore, the corresponding methyltransferase was not expressed from the *E. coli* chromosome.

**Fig. 1:**
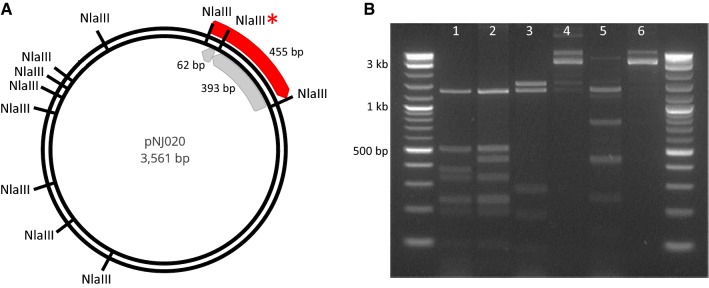
Confirmation of methyltransferase functionality in *E. coli*. **a** pNJ020 plasmid map with *Nla*III restriction sites, including *Nla*III* that overlaps with the GTCAT methylation site. When blocked by methylation, *Nla*III* is not cut by *Nla*III, resulting in a 455 bp band rather than 393 and 62 bp bands. **b** Agarose gel of restriction digests of plasmid pNJ020 isolated from *E. coli* Top10 (lanes 1, 3, and 5) and from methylating strain AG2006 (lanes 2, 4, and 6). Methylation at GTC**A**T is indicated by a band shift from 393 to 455 bp (lane 1 vs 2) or complete blockage of digestion by G**GC**C (lane 3 vs 4) and GCWGC (lane 5 vs 6)

The in vivo activity of Cthe_0519 and Phi3TI methyltransferases was determined by restriction enzyme digestion to examine the extent of methylation by our heterologously expressed methyltransferases. Many commercially available restriction enzymes are blocked by overlapping DNA methylation. Therefore, a restriction enzyme was chosen for each motif that either partially overlaps the motif of interest or, when possible, targets the exact same motif. If the plasmid is properly methylated, then the enzyme will be blocked by the methylation and no cutting will occur. For instance, restriction enzyme *Nla*III cuts unmethylated CATG, and a fraction of these *Nla*III sites will overlap with GTCAT (when the sequence GTCATG occurs) and will not be cut (Fig. 1a). For pNJ020, methylation of GTC**A**T by Cthe_0519 blocks *Nla*III-cutting between a 393 and 62 bp band, resulting in the generation of 455 bp band instead (Fig. 1a). Complete blockage of this cut site, and therefore compete methylation, was observed for Cthe_0519 (Fig. 1b, Lane 1, 2), where complete disappearance of the 393 bp band and generation of the new 455 bp band are observed. For the G**GC**C motif, restriction enzyme *Hae*III targets the same sequence, so we would expect complete blockage of restriction activity if methylation of this sequence occurs in *E. coli*. We linearized the plasmid with *Hin*dIII and digested with *Hae*III (Fig. 1). Unmethylated DNA (Lane 3) was completely digested, while Phi3TI methylation mostly blocked *Hae*III digestion (Lane 4), suggesting nearly complete methylation of G**GC**C by Phi3TI. For G**C**W**G**C methylation, enzyme *Tse*I targets the same motif, so the plasmid was linearized with *Hin*dIII and digested with *Tse*I. Unmethylated DNA (Lane 5) was completely digested, while Phi3TI methylation fully blocked *Tse*I digestion (Lane 6), suggesting complete methylation of G**C**W**G**W by Phi3TI.

### Methylated DNA allows transformation of *C. thermocellum* ATCC 27405

To determine if targeted DNA methylation would allow transformation of *C. thermocellum* ATCC 27405, we tested transformation efficiency using pNJ020. This plasmid contains 8 GTC**A**T, 7 G**C**W**G**C, 5 G**GC**C, 5 G**A**TC, and no CNC**A**NNNNNN**T**TC sites. Plasmid DNA was methylated by the control strain Top10 *Δdcm*, which only methylates GATC, and pNJ020 methylated with Cthe_0519 and Phi3TI, which methylates G**A**TC, GTC**A**T, G**GC**C (partially), and G**C**W**G**C, where all putative restriction systems should be evaded. The control transformation in which pNJ020 was only methylated with native *E. coli* Dam yielded no colonies, while the methylated plasmid yielded an average of 80 colony-forming units (CFU)/μg of plasmid DNA. Transformation of plasmid pAMG216 was also tested, which contains one Type I motif, and an average of 40 CFU/µg was observed (Table 2).

**Table 2:** Transformation efficiency of *C. thermocellum* ATCC 27405 using methylated plasmid DNA

Plasmid	Experiment			Average (CFU/µg)
	1	2	3	
pNJ020	27	62	151	80
pAMG216	14	20	85	40

No transformation was seen using control DNA without *C. thermocellum* methylation. Units are in colony-forming units (CFU) per microgram of plasmid DNA

## Discussion

Development of genetic systems in non-model microbes is a grand challenge for the study of both fundamental and applied microbiology. Here, we show a rational, systematic process for developing transformation methods in *C. thermocellum* ATCC 27405 by overcoming the native RM systems. This was done by first identifying the methylated motifs using high-throughput sequencing techniques, including the rarely used WGBS technique for identifying m^5^C motifs. Methylome analysis was followed by heterologous expression of targeted methyltransferases from the *E. coli* chromosome to mimic the *C. thermocellum* ATCC 27405 methylome. Plasmid DNA was passaged through this *E. coli* strain to methylate it, and functionality of the methylases was confirmed through restriction enzyme digest of the plasmid DNA. Properly methylated plasmid avoids the native RM systems and allowed transformation using commercially available equipment, which opens the door to metabolic engineering and the development of more advanced genetic tools. By enabling reliable transformation, the type strain of *C. thermocellum* can now be studied through genetic manipulation.

The first step to understanding and overcoming the native RM systems in *C. thermocellum* ATCC 27405 and other organisms is identifying the methylated motifs and the cognate methyltransferases. PacBio SMRT sequencing is the most commonly utilized method for microbial methylome analysis and can accurately identify m^6^A and m^4^C motifs. SMRT sequencing revealed three methylated motifs, and the corresponding RM systems were identified using REBASE. While SMRT sequencing is also able to detect m^5^C motifs, it does not always identify these motifs [37]. For example, the two m^5^C motifs in *C. thermocellum* ATCC 27405 were not discovered in the SMRT methylome analysis. Therefore, WGBS is a vital step to reliably determine the full methylome of a bacterial strain. Using WGBS on an Illumina platform, we were readily able to detect m^5^C motifs with less sequencing coverage. Recently, Oxford Nanopore sequencing has also been shown to detect DNA methylation [36]. As technologies advance, the simplicity of methylome analysis will likely increase.

Previous studies have expressed methyltransferases to methylate DNA prior to transformation, but functionality of these enzymes beyond examining the impact on transformation efficiency is rarely tested. Here, we tested functionality of the expressed methyltransferases through a restriction enzyme digestion assay in which restriction activity is blocked when the plasmid is methylated. While the Cthe_0519 methyltransferase fully methylated the plasmid DNA, the Phi3TI methyltransferase only partially methylated the DNA. By digesting plasmid DNA isolated from the *E. coli* methylation strain with an enzyme that overlaps with the methylated motif of interest, functionality can be easily determined by the percentage of DNA cut/uncut. This approach also unambiguously confirmed that Cthe_0519 methylates GTC**A**T.

An alternative approach to evading RM systems is to use plasmids that lack the targeted sequence. We used this approach for the putative Type I RM system (Cthe_1144–1145), where the motif was avoided using plasmid DNA (pNJ020) that does not contain the motif CNC**A**NNNNNN**T**TC. Thus, isolation of pNJ020 out of the *E. coli* methylation strain results in plasmid DNA that fully mimicked the ATCC 27405 methylome. While this approach can be helpful, it is not always possible to avoid or remove the target motifs. For instance, when the motif is short, such as a four-base recognition sequence, it may not be feasible to remove them all. Some motifs may also happen to be in critical parts of the sequence, such as when they are in the origin of replication or in a sequence being used for homologous recombination. Therefore, expression of methyltransferases in *E. coli* will likely continue to be an attractive approach for developing transformation tools in new organisms.

Interestingly, Cthe_1144–1145 seems to not be part of an active restriction system. It lacks a predicted restriction enzyme, and when transformation of a plasmid containing the corresponding Type I motif (pAMG216) was tested, there was not a substantial difference in transformation efficiency relative to pNJ020, suggesting that this system, indeed, lacks a restriction enzyme. The twofold difference in efficiency can likely be explained by the difference in plasmid size (pNJ020 is 3561 bp, while pAMG216 is 4700 bp), where the smaller plasmid has a greater number of plasmid molecules per microgram of DNA and may enter the cell more easily due to its smaller physical size.

We have demonstrated reproducible transformation of *C. thermocellum* strain ATCC 27405 and are now poised to improve these methods to increase frequency. Improved methylation by the Phi3TI methyltransferase will likely increase transformation efficiency. In addition, for reasons unknown, strain ATCC 27405 does not form a cell pellet during centrifugation as well as strain DSM 1313. Therefore, identifying growth conditions under which ATCC 27405 forms tight cell pellets would make competent cell preparation simpler and presumably increase competent cell concentration, potentially leading to increased efficiencies. However, all approaches for genetic manipulation in *C. thermocellum* to date have relied on use of replicating plasmids, even for gene deletions [29], so the full suite of *C. thermocellum* genetic tools is now available in strain ATCC 27405, even at the current transformation efficiency.

In conclusion, the combination of complete methylome analysis via both PacBio SMRT sequencing and WGBS and validated DNA methyltransferase expression allowed the rational development of transformation methods for *C. thermocellum* ATCC 27405. We anticipate that the approach for obtaining transformation demonstrated in this work may be applied to many other bacterial strains, especially in new, non-model organisms.

## Electronic supplementary material

Below is the link to the electronic supplementary material.

Supplementary material 1 (GB 12 kb)

Supplementary material 2 (GB 13 kb)

Supplementary material 3 (GB 11 kb)

Supplementary material 4 (GB 8 kb)
